# Endoscopic submucosal dissection of early gastric cancer in the gastric fundus: challenges and techniques

**DOI:** 10.1055/a-2602-3045

**Published:** 2025-06-13

**Authors:** Rafael Prado Pessoa, Laís Martins Magalhães Almeida, Caroline Assis Aleixo Chaves, Lucas Gallo de Alvarenga Mafra, Bernardo Ferreira de Paula Ricardo, Nelson Tomio Miyajima, Rodrigo Roda

**Affiliations:** 1 Endoscopy Division, Rede Mater Dei de Saúde – Unidade Santo Agostinho, Belo Horizonte, Brazil; 2 223018 Department of Pathology, Rede Mater Dei de Saúde, Belo Horizonte, Brazil; 3 Endoscopy Division, Hospital das Clínicas da Universidade de São Paulo, São Paulo, Brazil; 4 219764 Endoscopy Division, Hospital das Clinicas da Universidade Federal de Minas Gerais, Belo Horizonte, Brazil

Endoscopic submucosal dissection (ESD) of tumors located in the gastric fundus is technically challenging. The wall is thin and has a rich vascularization, which increases the risk of perforation and bleeding 1 . The procedure is mostly performed in retroflexion, and the tip of the endoscope has limited reach.

In this video ( Video 1 ), we presented a case of a 73-year-old patient with upper gastrointestinal endoscopy showing an elevated superficial lesion (0–IIa) measuring 30 mm × 20 mm, in the gastric fundus ( Fig. 1 ). Biopsies showed high-grade dysplasia.

Endoscopic submucosal dissection of early gastric cancer in the gastric fundus using the underwater approach and an external traction technique.Video 1

**Fig. 1:**
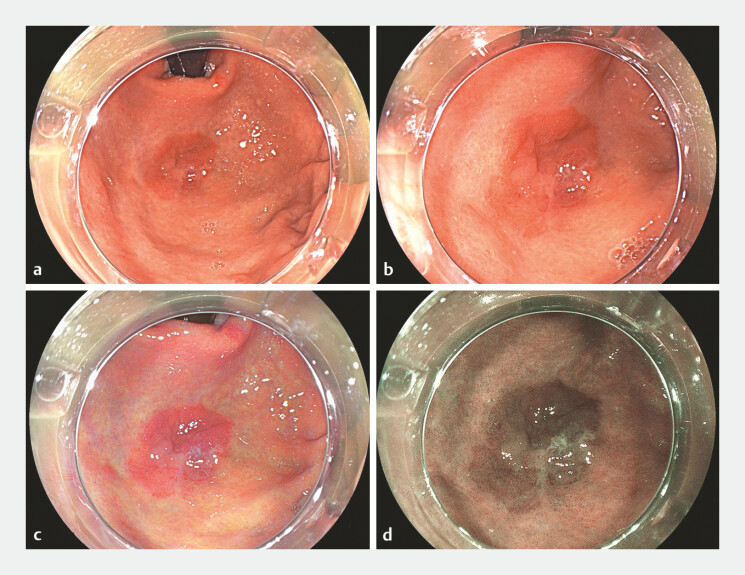
Elevated superficial lesion (Paris 0–IIa) measuring approximately 30 mm × 20 mm, in the gastric fundus. Targeted biopsies showed high-grade dysplasia. **a** and **b** White light aspect of the lesion. **c** LCI. **d** BLI. Abbreviations: BLI, blue laser imaging; LCI, linked color imaging.

The procedure was performed with an optical magnification gastroscope (EG-760Z, Fujifilm Medical). The knife used was an injectable needle knife (ORISE ProKnife; Boston Scientific).

Due to the gastric fundus thin wall, it was possible to visualize the visceral fat through the wall. At two points, the dissection was deepened into the muscular layer, without complete perforation. The underwater technique was then performed to avoid over-distension of the organ, an undesirable distance of the resection area and to position the tip of the knife more parallel to the muscular layer 2 ( Fig. 2 ).

**Fig. 2:**
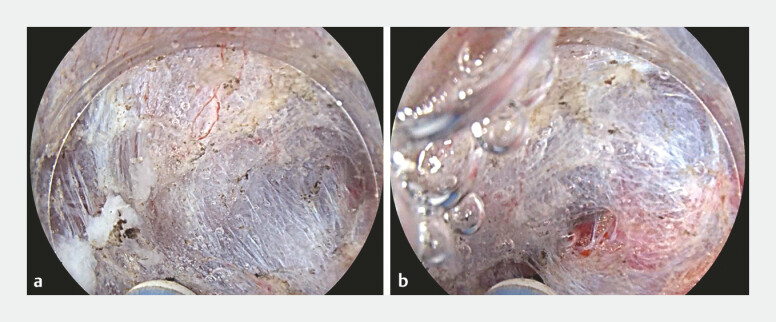
Underwater approach. The tip of the knife is almost parallel to the muscle layer, which makes dissection safer against perforation.

External traction method with clip and snare was also used 3 ( Fig. 3 ). This traction technique allows you to pull or push the snare, which was attached to the clip, applying the appropriate tension to safely expose the submucosa.

**Fig. 3:**
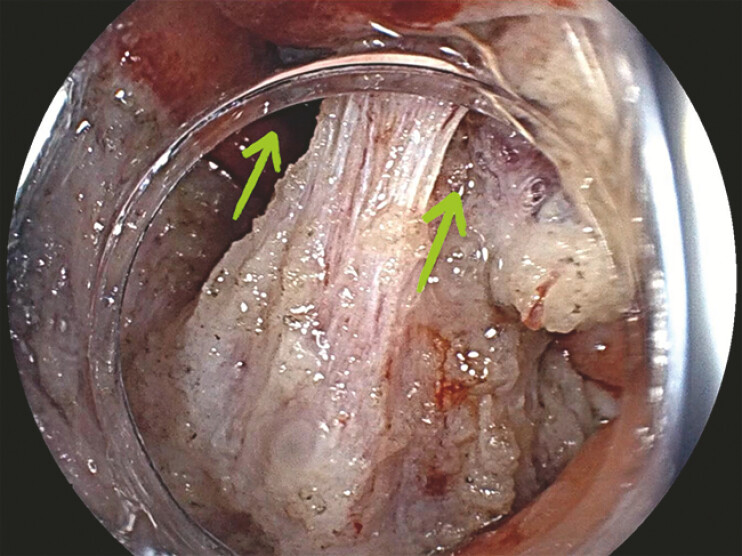
External traction technique using clip and snare. This traction technique allows you to pull or push the snare, which was attached to the clip, applying the appropriate tension to safely expose the submucosa.

After complete resection of the lesion, it was placed two clips over the submucosa and then, complete closure of the ulcer area ( Fig. 4 ).

**Fig. 4:**
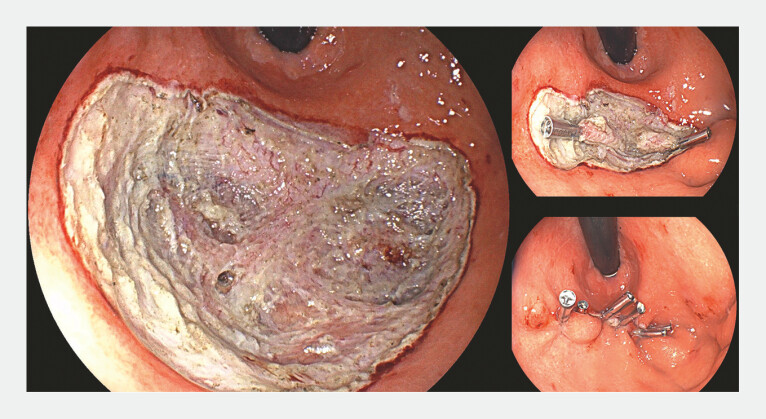
Final aspect of the procedure. Two clips were applied into the dissection layer, followed by the complete closure of the ulcer.

Regarding histopathology, the resection was classified as endoscopic curability C-2 (eCuraC-2) ( Fig. 5 ). Therefore, the patient must undergo surgical completion 4 .

**Fig. 5:**
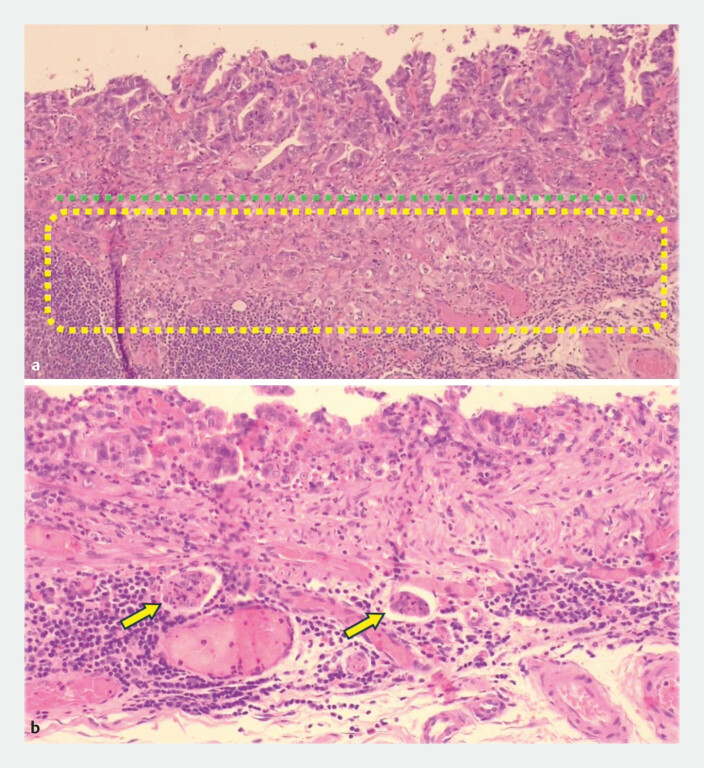
**a** The green dotted line corresponds to the muscularis mucosae. The content of the yellow rectangle corresponds to the invasion of the submucosa. **b** The arrows correspond to the angiolymphatic emboli.

With the case presented, we can conclude that the endoscopist must be prepared to use techniques such as external traction and underwater dissection for the viability of technically challenging ESD’s. The monobloc resection ensured accurate staging and reinforced surgical indication.

Endoscopy_UCTN_Code_TTT_1AO_2AG_3AD

## Notes

Endoscopy E-Videos https://eref.thieme.de/e-videos
*E-Videos* is an open access online section of the journal *Endoscopy* , reporting on interesting cases and new techniques in gastroenterological endoscopy. All papers include a high-quality video and are published with a Creative Commons CC-BY license. Endoscopy E-Videos qualify for HINARI discounts and waivers and eligibility is automatically checked during the submission process. We grant 100% waivers to articles whose corresponding authors are based in Group A countries and 50% waivers to those who are based in Group B countries as classified by Research4Life (see: https://www.research4life.org/access/eligibility/ ). This section has its own submission website at https://mc.manuscriptcentral.com/e-videos .
